# Support for a point-of-sale cigarette display ban among smokers: findings from the international tobacco control (ITC) Netherlands survey

**DOI:** 10.1186/s12889-018-5666-4

**Published:** 2018-06-15

**Authors:** Dirk-Jan A. van Mourik, Math J. J. M. Candel, Gera E. Nagelhout, Marc C. Willemsen, Geoffrey T. Fong, Karin Hummel, Bas van den Putte, Hein de Vries

**Affiliations:** 1 Department of Health Promotion, Maastricht University (CAPHRI), PO Box 616, 6200 MD Maastricht, the Netherlands; 2 Department of Methodology and Statistics, Maastricht University (CAPHRI), Maastricht, the Netherlands; 3 Department of Family Medicine, Maastricht University (CAPHRI), Maastricht, the Netherlands; 4 IVO Addiction Research Institute, The Hague, the Netherlands; 5 Netherlands Expertise Center for Tobacco Control (NET), Trimbos Institute, Utrecht, the Netherlands; 6 Department of Psychology, University of Waterloo, Waterloo, Canada; 7 Ontario Institute for Cancer Research, Toronto, Canada; 8 School of Public Health and Health Systems, University of Waterloo, Waterloo, Canada; 9 Department of Communication, University of Amsterdam (ASCoR), Amsterdam, the Netherlands

**Keywords:** Support, Tobacco display ban, Point of sale, The Netherlands, Smokers

## Abstract

**Background:**

Displaying tobacco products at point-of-sale (PoS) has become an important marketing strategy for the tobacco industry. This study was designed to (1) examine how support for a PoS cigarette display ban changed among Dutch smokers between 2010 and 2015 and (2) identify the variables that predict support among smokers for a PoS cigarette display ban.

**Methods:**

Longitudinal data from six annual survey waves (2010-2015) from the International Tobacco Control (ITC) Netherlands Survey were analyzed. The sample consisted of between 1279 and 1800 smokers per year. Smokers were asked whether they supported a complete ban on displays of cigarettes inside shops and stores.

**Results:**

Support for a PoS cigarette display ban increased from 28.9% in 2010 to 42.5% in 2015 (OR = 1.40, *p* < 0.001). A multiple logistic regression analysis revealed that support for a PoS display ban of cigarettes was more likely among smokers who had more knowledge about the health risks of smoking (OR = 3.97, *p* < 0.001), believed smoking-related health risks to be severe (OR = 1.39, *p* < 0.001), had a more positive attitude towards quitting smoking (OR = 1.44, *p* = 0.006), reported stronger social norms to quit smoking (OR = 1.29, *p* = 0.035), had a higher self-efficacy for quitting smoking (OR = 1.31, *p* = 0.001), and had stronger intentions to quit smoking (OR = 1.23, *p* = 0.006).

**Conclusions:**

This paper showed that support for a PoS display ban of cigarettes increased among smokers in the Netherlands over the years. To further increase support, educational campaigns about the dangers of smoking, and campaigns that encourage quitting may be needed.

## Background

As countries take action to reduce tobacco advertising, promotion, and sponsorship (TAPS), the tobacco industry has fewer opportunities to promote their products. Displaying tobacco products at point-of-sale (PoS) has become one of the most important remaining tools for the tobacco industry to communicate with current and potential customers [1–5], increasing the importance of PoS tobacco display bans to reduce TAPS.

Internal documents of the tobacco industry suggested that tobacco displays are used to shape positive attitudes and beliefs about tobacco brands and products [6]. Displaying tobacco products at PoS can act as a cue to smoke [7–11], even among people who try to avoid smoking [12]. Research has also shown that exposure to PoS tobacco displays increases susceptibility for smoking uptake among youth [4, 13, 14]. Restrictions on PoS tobacco displays can lead to fewer display recalls [15] and may help to denormalise smoking [16].

The World Health Organization (WHO) Framework Convention on Tobacco Control (FCTC) calls on the 180 parties (179 countries and the European Union) to implement PoS tobacco display bans [17]. Several jurisdictions including Canada, Iceland, Norway, Finland, United Kingdom, and Ireland have introduced a PoS tobacco display ban but global progress in this domain has been slow. In the Netherlands, a ban on PoS tobacco displays in supermarkets is planned for 2020. For other points of sale such as gas stations, convenience stores, drug stores, bars and cafes, evening shops, and kiosks, a ban on PoS tobacco displays is planned for 2022 [18]. A PoS tobacco display ban might be especially effective in the Netherlands where the number of inhabitants per PoS is low compared to other countries [19].

High levels of public support for tobacco control measures, particularly among smokers, may be an important condition for the adoption of these measures by the government [20]. High levels of public support among smokers for a PoS tobacco display ban could prevent resistance that could endanger the implementation in 2020 and the continuation of this ban. This is of utmost importance in the Netherlands, where tobacco control policies have been reversed and delayed in the past [21]. In 2014, 60% of all Europeans and 56% of the Dutch population supported keeping tobacco products out of sight at PoS [22]. While non-smokers generally are more likely to support tobacco control measures than smoker [23, 24], many smokers are also supportive. In Ireland, 67% of the non-smokers and 63% of the smokers were supportive of a PoS display ban of cigarette and tobacco packs after the implementation of this tobacco control measure [15]. A study from Canada found that the levels of support for a ban on PoS displays of cigarettes ranged between 55 and 82% (in Canadian provinces) among adult smokers [25]. These studies show reasonable levels of support among smokers for a ban on PoS tobacco displays but studies examining possible predictors of this support remain limited.

Identifying these predictors may help policy makers to increase support levels. Data from the International Tobacco Control (ITC) Canada Survey revealed that smokers with higher intention to quit were more likely to support a PoS cigarette display ban [25]. This study only focused on intention to quit smoking and socio-demographic characteristics but did not include smoking cessation related beliefs. The current study examines which factors may predict support for a PoS tobacco display among Dutch smokers.

As a multitude of factors may be associated with support for PoS display bans, we used two integrated behavior change models: the ITC Conceptual Model [26], and the Integrated Change Model (I-Change Model) [27]. The ITC Conceptual Model is used to explain how tobacco control measures might work based on a combination of health communication theories and existing psychological models [26]. The I-Change Model can be used to explain overt (directly observable), and covert (not immediately observable) health behaviors [27] such as supporting a PoS display ban. Based on these models we identified two groups of factors: (1) socio-demographic characteristics (age, gender, and educational level), and (2) smoking cessation related beliefs such as awareness (knowledge, cues such as noticing anti-tobacco information, and risk perception), motivation factors (attitude, social norms, and self-efficacy for quitting smoking), and intention to quit smoking.

Lowly educated smokers have lower intentions to quit smoking [28, 29]. Since intention to quit smoking predicts support for a PoS tobacco display ban [25], the question arises whether lowly educated smokers are less often supportive. Insight into educational differences may enable policy makers to differentiate in the educational approach on tobacco display bans. Therefore, this study aims to examine differences between low, moderate, and high educated smokers in predictors and trends of support for a PoS tobacco display ban.

This study aims to answer the following research questions: (1) Did support among Dutch smokers for a PoS cigarette display ban change over time from 2010 to 2015? (2) Which factors predict support among smokers for a PoS cigarette display ban? (3) Are the findings from the first two research questions different for low, moderate, and high educated smokers?

## Methods

### Sample

Longitudinal data were obtained from the ITC Netherlands Survey. The surveys were administrated via the internet by the research firm Kantar Public (previously TNS NIPO) which used a quota sample of respondents from a probability-based web database to retrieve a representative sample of Dutch smokers aged 15 years and older [30]. Respondents were categorized as smokers if they were currently smoking at least monthly and if they had smoked at least 100 cigarettes in their lifetime [31]. Sampling weights and tailored replenishment samples ensured representativeness by compensating for attrition effects [32]. Respondents received incentives for participation in the form of points for each answered question, which could be exchanged for gift certificates.

**Fig. 1:**
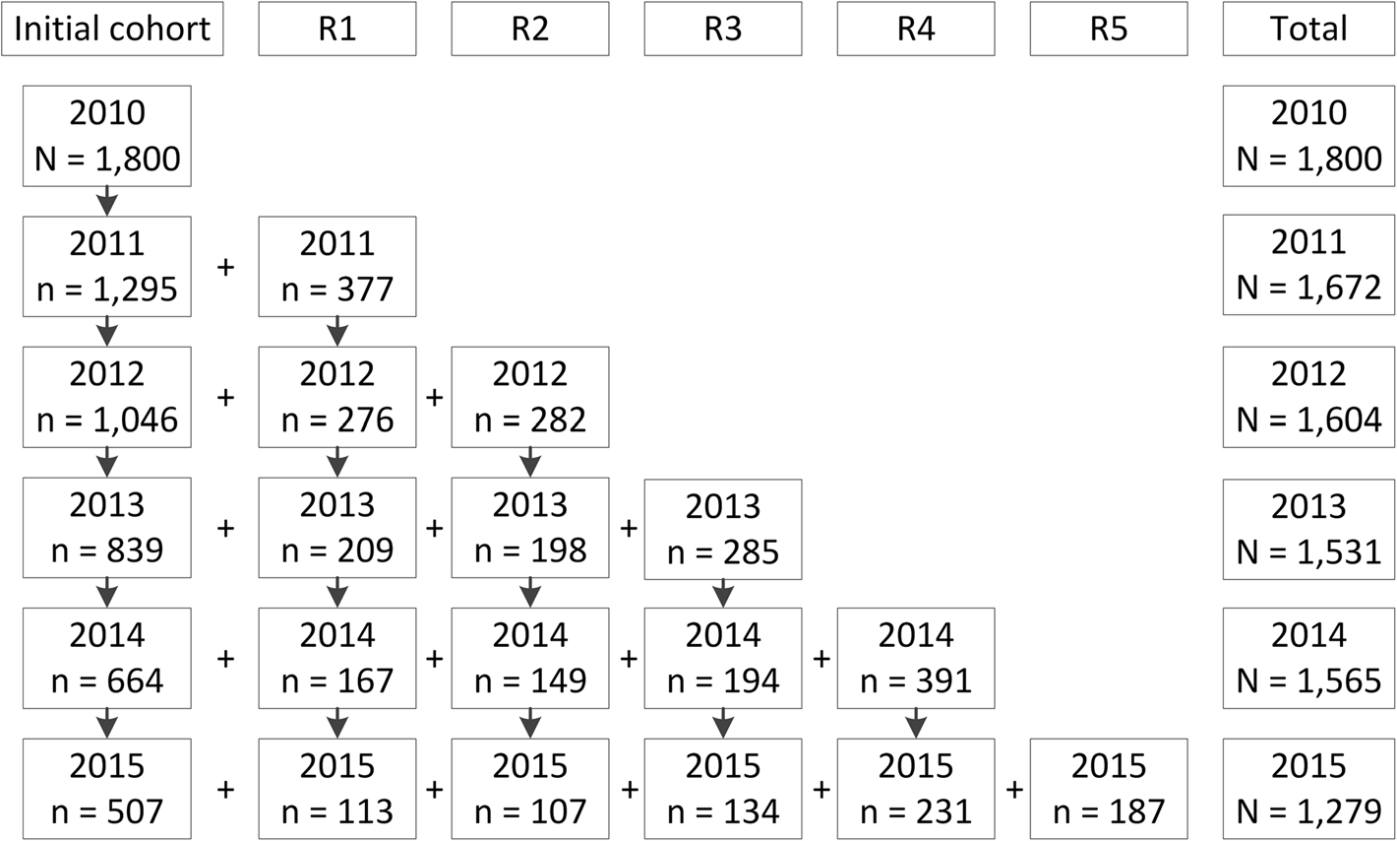
Flowchart of the International Tobacco Control Netherlands Survey recruitment of smokers*. *R = Replenishment

For our analyses, we used data from survey wave 4 (May to June 2010; *N* = 2060), wave 5 (May to June 2011; *N* = 2101), wave 6 (May to June 2012; *N* = 2022), wave 7 (May to June 2013; *N* = 1970), wave 8 (May to June 2014; *N* = 2008), and wave 9 (November to December 2015; *N* = 1720). Attrition ranged from 17.1 to 23.9% between survey waves. We excluded quitters from our analyses. Exclusion due to smoking cessation ranged from 12.6 to 25.6% between survey waves. Figure 1 shows the number of smokers of the initial cohort and of the replenishment samples that remained in the study for each wave, leading to a total number of smokers per survey wave. To answer research question 1, all smokers from survey waves 4-9 were included in the analyses.

In the analyses for research question 2 we used the two most recent survey waves (waves 8 and 9). Respondents were included in the analyses if they were categorized as a smoker in both waves. In wave 9, 1017 of 1565 smokers from wave 8 participated. Respondents were excluded if they had more than five missing values on the independent variables (out of 22 variables) or if they had not filled in the outcome variable on wave 9. This left 844 smokers eligible for the analyses of research question 2.

### Outcome variable

Support for a PoS cigarette display ban was measured by asking ‘Do you support complete bans on displays of cigarettes inside shops and stores?’ [25]. This measure used a three-point scale with the response options (0) not at all, (1) somewhat, and (2) a lot. This variable was dichotomized for the analyses by combining the last two response options.

### Socio-demographic variables

Respondents were asked whether they were (1) male or (2) female and were classified in one of the following age groups: (1) 15-24, (2) 25-39, (3) 40-45, or (4) 55 years and older. Education was divided into three categories: (1) low (Primary education and lower pre-vocational secondary education), (2) moderate (Middle pre-vocational secondary education and secondary vocational education), and (3) high (Senior general secondary education, (pre-) university education and higher professional education).

### Awareness variables

Knowledge about the health risks of smoking was measured by asking eight questions. The following format was used: ‘The following are a few health effects and diseases. Based on what you know or believe, does smoking cause ...’ [33]. This question was asked for heart disease, impotence in male smokers, lung cancer, blindness, mouth and throat cancer, stroke, lung-cancer in non-smokers from secondhand smoke, and heart disease in non-smokers from secondhand smoke (α in wave 8 = 0.84). Respondents could answer with (1) yes, (0) no, and (0) don’t know.

Noticing anti-tobacco information was assessed by asking if respondents had noticed advertising or information about the dangers of smoking or advertising that encouraged quitting in the last 6 months [34]. There were five answering categories ranging between (1) never and (5) very often.

Risk perception was assessed by measuring perceived susceptibility and perceived severity. Perceived susceptibility was obtained by the question ‘If you continue to smoke the amount you do now, how likely do you think it is that you will develop lung cancer in the future?’ The scale ranged between (1) very low and (5) very high. Additionally, all smokers were asked about perceived severity via the question ‘If you develop lung problems due to smoking, how serious would you find this?’ The scale ranged between (1) not at all serious and (5) extremely serious.

### Motivational variables

Attitude towards quitting smoking was asked via the questions ‘If you quit smoking within the next 6 months, this would be…’: (1) very foolish to (5) very wise and sensible, (1) very disagreeable to (5) very agreeable, and (1) very negative to (5) very positive. The three answers were summed into one attitude score (α in wave 8 = 0.83).

Social norms for quitting smoking were assessed by asking ‘Thinking about the people who are important to you, how do you think most of them would feel about your quitting smoking within the next 6 months?’ [35]. Based on a five-point scale respondents could (1) strongly disapprove to (5) strongly approve.

Self-efficacy to quit smoking was measured by asking how sure they were to succeed if they decided to give up smoking completely in the next 6 months [29]. This was measured on a five-point scale ranging from (1) not at all sure to (5) extremely sure. Smokers were also asked how easy or hard it would be to quit smoking if they wanted to. Answering categories ranged between (1) extremely difficult and (5) not at all difficult. The two answers were summed into one self-efficacy score (α in wave 8 = 0.72).

### Intention to quit smoking variable

Intention to quit smoking was measured by asking if the respondents were planning to quit smoking within the next 6 months [35, 36]. A five-point scale ranging between (1) very unlikely and (5) very likely was used.

### Smoking-related variables

Smoking-related covariates were level of addiction to tobacco, and ever having tried to quit smoking. The Heaviness of Smoking Index (HSI) was used to measure the level of addiction to tobacco. The HSI is based on the time before smoking the first cigarette of the day (61 + min, 31-60 min, 6-30 min, 5 min or less) and on the number of cigarettes smoked per day (0-10, 11-20, 21-30, 31+). This measure ranges between 0 and 6, with a higher score indicating a higher level of addiction to tobacco [37]. Also, the respondents were asked whether they had ever tried to quit smoking, (1) yes or (2) no.

### Statistical analysis

Data were analyzed using the statistical software program SPSS 23. All statistical estimates and tests presented were weighted for gender and age [38].

Trends in outcome measure (research question 1) were tested with Generalized Estimating Equations (GEE) analyses [39], while controlling for gender, age, ever having made a quit attempt, education, and level of addiction to tobacco. Missing data values on these variables were imputed multiple times using the full conditional specification method (with linear regression for scalar covariates) [40]. The number of imputations was set according to the percentage of cases that were incomplete [41]. Moreover, starting from this number of imputations, we systematically increased the number of imputations by 10 until results hardly differed. This resulted in 80 imputations. Also, we adjusted for the time a respondent participated in the cohort (time-in-sample) since this may influence responses [42]. The dependent variable was dichotomous and therefore the binomial distribution and the logit link was used [43]. Survey wave was the repeated measure variable. The interaction between educational level and survey wave was assessed in a separate analysis (research question 3).

T-tests and Chi-square analyses were run to test differences in independent variables (measured in 2014) between supportive and non-supportive smokers of a cigarette display ban (measured in 2015). The associations between independent variables on wave 8 with support for a PoS cigarette display ban on wave 9 (research question 2) were examined by performing multiple logistic regression analyses, while controlling for gender, education and age. To examine total effects of predictor variables, we should not correct for possible mediators or descending proxies thereof [44]. For this reason, the variables were added in successive steps: first the awareness variables were entered (knowledge about the health risks of smoking, perceived severity, perceived susceptibility, and noticing anti-tobacco information), second the motivational variables (attitude, social norms and self-efficacy), and in the last step intention to quit smoking. The analysis results for the variables as reported, are those for the step in which they were first entered into the analysis model. Only 477 out of the eligible 844 respondents completed all independent variables. For the remaining 367 respondents missing values were filled in by multiple imputation. The 884 subjects available for the analysis therefore contained no persons with missing values on the outcome, but with possibly missing values on predictor variables that were filled in by multiple imputation. This method improves the statistical power by increasing the sample size for the multiple logistic regression [40]. Following the same procedure as for the trend analysis, the number of imputations was set at 100. The imputed values for the outcome variable were not employed in the analyses, since excluding cases with missing values on the outcome variable yields more stable estimates [45]. Because the variables education and age each were represented in the analyses through multiple dummy variables and SPSS provided only *p*-values for these separate dummy variables when analyzing multiple imputed datasets, a correction for multiple testing was applied. More specifically, for all tests involving the variables education and age, the Holm correction was applied to the significance level α = 0.05 [46]. Interactions between educational level and independent variables were added in a separate regression analysis to determine whether there were differences between educational levels in the predictors for support for a PoS cigarette display ban (research question 3). This analysis was also done and reported with the same steps as delineated above, but then also adding each time the interaction terms with education.

## Results

### Sample characteristics

Table 1 shows characteristics of smokers between 2010 and 2015. The smokers’ educational level increased over the years.

**Table 1:** Sample characteristics of smokers between 2010 and 2015^a^

	2010	2011	2012	2013	2014	2015
Gender						
Male (%)	53.6	54.4	54.1	52.0	50.7	54.0
Female (%)	46.4	45.6	45.9	48.0	49.3	46.0
Age group						
15-24 years (%)	12.7	22.8	13.7	11.5	11.1	15.5
25-39 years (%)	27.5	28.9	23.5	23.7	23.8	24.1
40-54 years (%)	32.3	25.1	32.1	32.2	29.8	27.9
55 years and older (%)	27.5	23.2	30.7	32.6	35.3	32.6
Educational level						
Low (%)	36.2	30.2	31.8	29.1	26.1	24.7
Moderate (%)	41.9	45.9	44.9	46.1	43.7	42.4
High (%)	21.9	24.0	23.3	24.8	30.2	33.0
Level of addiction to tobacco						
0 to 1 (%)	29.2	30.8	28.0	28.7	29.4	31.7
2 to 4 (%)	64.4	58.1	64.5	64.4	64.5	62.1
5 to 6 (%)	6.4	11.2	7.5	6.9	6.0	6.2
Ever tried quitting smoking						
Yes (%)	65.1	60.7	60.9	60.2	57.8	64.1
No (%)	34.9	39.3	39.1	39.8	42.2	35.9

^a^Estimates were weighted for gender and age

### Support for a PoS cigarette display ban between 2010 and 2015

Table 2 shows that support for a PoS display ban of cigarettes increased from 28.9% in 2010 to 42.5% in 2015. A GEE analysis confirmed the overall linear trend (Odds Ratio (OR) = 1.40, 95% Confidence interval (CI): 1.25, 1.58).

**Table 2:** Percentages of smokers by educational level and wave who support a PoS cigarette display ban^a^

	2010	2011	2012	2013	2014	2015
Total group (%)	28.9	34.6	34.4	36.4	37.7	42.5
Low educational level (%)	24.6	34.8	33.7	38.4	37.6	38.6
Moderate education level (%)	30.5	33.1	31.0	34.5	36.7	38.2
High educational level (%)	30.8	37.4	41.2	38.5	39.1	50.9

^a^Estimates were weighted for gender and age

A separate GEE analysis including interaction terms of wave by educational level revealed that the support for a PoS cigarette display ban increased less among moderately educated smokers than among highly educated smokers (OR of interaction = 0.87, 95% CI: 0.78, 0.98, *p* = 0.011 < α_adjusted_ = 0.017). This result can also be seen in Table 2 that shows that the support among moderately educated smokers increased from 2010 to 2015 by 7.6% whereas the support for highly educated smokers increased by 20.1%. No significant interactions were found between wave and low versus moderate education (OR = 1.07, 95% CI: 0.97, 1.18, *p* = 0.155 > α_adjusted_ = 0.025), nor between wave and low versus high education (OR = 0.94, 95% CI: 0.84, 1,05, *p* = 0.238 > α_adjusted_ = 0.05).

### Predictors of support for a PoS cigarette display ban

The bivariate analyses from Table 3 show that smokers who support a PoS cigarette display had more knowledge about the health risks of smoking, had a higher perceived severity of smoking-related lung problems, had a more positive attitude towards quitting smoking, perceived stronger social norms for quitting, had a higher self-efficacy for quitting smoking, and a higher intention to quit smoking.

**Table 3:** Bivariate t-tests and Chi-square tests of predictor variables in 2014 and support in 2015^a,b^

	Support		
	Yes (*n* = 343)	No (*n* = 501)	T-value or χ^2^
Socio demographics			
Gender			
Male (%)	40.2	59.8	χ^2^ = 0.43
Female (%)	40.9	59.1	*p* = 0.836
Age group			
15-24 years (%)	34.1	65.9	χ^2^ = 3.91
25-39 years (%)	45.8	54.2	*p* = 0.271
40-54 years (%)	39.1	60.9	
55 years and older (%)	40.4	59.6	
Educational level			
Low (%)	38.8	61.2	χ^2^ = 0.77
Moderate (%)	39.3	60.7	*p* = 0.681
High (%)	43.3	56.7	
Awareness variables			
Knowledge health risks of smoking (mean, SD)	0.65 (0.27)	0.54 (0.30)	*t* = −5.62, *p* < 0.001
Noticing anti-tobacco information (mean, SD)	2.41 (0.96)	2.29 (0.95)	*t* = −1.57, *p* = 0.116
Perceived susceptibility (mean, SD)	3.14 (0.96)	3.09 (0.84)	*t* = −0.82, *p* = 0.413
Perceived severity (mean, SD)	3.54 (1.03)	3.16 (0.99)	*t* = −5.42, *p* < 0.001
Motivational variables			
Attitude towards quitting smoking (mean, SD)	4.21 (0.70)	3.86 (0.79)	*t* = −6.80, *p* < 0.001
Social norms for quitting smoking(mean, SD)	4.41 (0.74)	4.10 (0.77)	*t* = −5.73, *p* < 0.001
Self-efficacy for quitting smoking (mean, SD)	2.45 (1.07)	2.23 (1.00)	*t* = − 2.95, *p* = 0.003
Intention to quit smoking (mean, SD)	2.77 (1.15)	2.32 (1.06)	*t* = − 5.85, *p* < 0.001

^a^Estimates were weighted for gender and age

^b^Imputed data

The results from the multiple logistic regression (Table 4) revealed that support for a PoS cigarette display ban was significantly associated with more knowledge about the health risks of smoking, a higher perceived severity, a more positive attitude towards quitting smoking, stronger social norms for quitting smoking, a higher self-efficacy for quitting smoking, and a higher intention to quit smoking.

**Table 4:** Multiple logistic regression analysis of predictors associated with support for a PoS cigarette display ban (*N* = 844, where *N* = 343 for the supportive group, *N* = 501 for the non-supportive group)^a,b,c^

	OR	95% CI
Socio demographics		
Gender		
Male	0.99	
Female	1.01	(0.88, 1.16)
Age group		
25-39 years vs.15-24 years (ref)	1.59	(0.92, 2.74)
15-24 years vs. 55+ (ref)	0.77	(0.46, 1.27)
25-39 years vs. 55+ (ref)	1.22	(0.84, 1.77)
40-54 years vs. 15-24 years (ref)	1.24	(0.79, 2.14)
40-45 years vs. 25-30 years (ref)	0.78	(0.53, 1.14)
40-45 years vs. 55+ (ref)	0.95	(0.71, 1.27)
Educational level		
Low vs. high (ref)	0.88	(0.60, 1.29)
Moderate vs. low (ref)	1.00	(0.70, 1.43)
Moderate vs high (ref)	0.88	(0.64, 1.23)
Awareness variables		
Knowledge health risks of smoking	3.97***	(2.25, 7.00)
Noticing anti-tobacco information	1.12	(0.96, 1.32)
Perceived susceptibility	0.91	(0.74, 1.11)
Perceived severity	1.39***	(1.20, 1.61)
Motivational variables		
Attitude towards quitting smoking	1.44**	(1.11, 1.87)
Social norms for quitting smoking	1.29*	(1.02, 1.64)
Self-efficacy for quitting smoking	1.31**	(1.12, 1.53)
Intention to quit smoking	1.23**	(1.06, 1.43)

^a^Estimates were weighted for gender and age

^b^Imputed data

^c^Associations are between independent variables on survey wave 8 and support on survey wave 9

*p < 0.05; **p < 0.01; ***p < 0.001

*OR* Odds ratio, *CI* Confidence interval, *ref* Reference category

A separate analysis including interaction terms with educational level and all independent variables did only show almost significant interactions. There was a nearly significant interaction between attitude towards quitting smoking and moderate versus high education (OR of interaction = 0.49, 95% CI: 0.25, 0.96, *p* = 0.038 > α_adjusted_ = 0.025), as well as a nearly significant interaction between attitude towards quitting smoking and moderate versus low education (OR of interaction = 0.46, 95% CI: 0.24, 0.90, *p* = 0.024 > α_adjusted_ = 0.017). Attitude towards quitting smoking was a stronger predictor for high educated smokers (OR = 2.07, *p* = 0.008, 95% CI: 1.21, 3.55), and for low educated smokers (OR = 2.10, *p* = 0.006, 95% CI: 1.23, 3.57) as compared to moderate educated smokers (OR = 0.97, *p* = 0.886, 95% CI: 0.64, 1.46).

## Discussion

Despite recommendations from the WHO dating back to 2003, the Dutch government only recently (January 2017) decided to implement a PoS cigarette display ban. In this paper we examined predictors of, and trends in support for this tobacco control measure among Dutch smokers.

The first research question was whether there were changes in support for a PoS cigarette display ban among Dutch smokers over time. We found a significant increase in support among smokers between 2010 (28.9%) and 2015 (42.5%). This is consistent with findings from other research that tobacco control measures are also popular among smokers, and support tends to increase over time especially after measures have been implemented [15, 47]. If this trend continues, it seems like a matter of time before the majority of Dutch smokers support this tobacco control measure. The increase in support for a PoS cigarette display ban may also be consequence of a general societal denormalisation of smoking, which is indicated by an increasing perceived societal disapproval of smoking [48].

The second research question aimed to identify factors that predict support among smokers for a PoS cigarette display ban. In line with previous research [25], a higher intention to quit smoking predicted support for a PoS display ban of cigarettes. The theory of self-control [49] provides a possible explanation for why smokers with a high intention to quit smoking support this tobacco control measure. A cigarette display ban may be perceived as a welcome external limit on future behavior (smoking) since leaving tobacco products out of sight at PoS will prevent smokers to get tempted to start smoking again. This may help smokers who want to quit doing this successfully. This explanation can also be used to explain the other predictors we found, since intention to quit smoking is associated with more knowledge about the health risks of smoking [50], more perceived severity [51], a more positive attitude towards quitting smoking [52], stronger social norms about quitting smoking [35], and higher self-efficacy for quitting smoking [53]. Smokers may thus be aware that exposure to PoS tobacco displays obstructs smokers to decrease or quit smoking [7–12]. Knowledge, perceived severity, attitude, social norms, and self-efficacy were independent predictors for support and should therefore be considered as important points of engagement for future campaigns.

The third research question was whether the findings from the first two research questions differed for smokers with low, moderate and high educated levels. First, support for a PoS cigarette display ban was higher and increased more among the highly educated group than among the moderately educated group. This could again be explained by the fact that less educated smokers tend to have lower intentions to quit [28, 29]. Second, we found that attitude towards quitting was a stronger predictor for high, and low educated smokers as compared to moderate educated smokers.

### Limitations

Several limitations should be taken into account when interpreting the results. First, the sample differed over the years on age, educational level, level of addiction to tobacco, and ever having made a quit attempt which may indicate that the sample is not entirely representative of the Dutch population of smokers. Therefore, we adjusted for these sample characteristics in the analyses and applied weights. Second, to cope with the high number of missing values we applied multiple imputation procedures to increase the statistical power. There is never complete certainty about the correctness of the imputation model but a rather substantial set of variables was used in this model and care was taken to take a large number of imputations to maximize the efficiency of the pooled estimates. Third, the measurements of wave 9 took place from November to December 2015, whereas in prior years measurements took place from May to June. This difference can give a somewhat distorted image of the trends in support. Fourth, this paper addressed support for a ban of cigarette displays. Since the ITC Netherlands survey did not include a question on support for a complete ban of tobacco displays, we could not study a display ban of other tobacco or nicotine products.

### Implications for policy

To increase support further, educational campaigns may focus on explaining the upcoming PoS tobacco display ban as well as on improving knowledge about the health risks of smoking, increasing perceived severity, stimulating a more positive attitude towards quitting smoking, changing social norms about quitting smoking, increasing self-efficacy for quitting smoking, and stimulating quitting. Such educational campaigns may be especially important in the Netherlands where the knowledge and general concern about the health risks of smoking and secondhand smoke in smokers is low compared to other countries [48].

## Conclusions

Support among Dutch smokers for a PoS cigarette display ban increased between 2010 and 2015, and increased faster among highly educated smokers than among moderately educated smokers. The findings from the present study showed that predictors of support among smokers for a PoS cigarette display ban were more knowledge about the health risks of smoking, higher perceived severity, more positive attitude towards quitting smoking, stronger social norms to quit smoking, higher self-efficacy for quitting smoking, and stronger intentions to quit smoking. To increase support, educational campaigns may focus on improving knowledge about the health risks of smoking, and encouraging quitting.

## Abbreviations

CI: Confidence interval
FCTC: Framework Convention on Tobacco Control
GEE: Generalized estimating equations
I-Change Model: Integrated change model
ITC: International Tobacco Control
OR: Odds ratio
PoS: Point-of-sale
TAPS: Tobacco advertising, promotion, and sponsorship
WHO: World Health Organization
